# Mapping Global Research Landscapes of Acupuncture for Diabetes Mellitus: A 20-Year Bibliometric Study (2004–2024)

**DOI:** 10.3390/healthcare13192468

**Published:** 2025-09-29

**Authors:** Tianyu Gu, Yuhan Nie, Huayuan Yang

**Affiliations:** School of Acupuncture-Moxibustion and Tuina, Shanghai University of Traditional Chinese Medicine, Shanghai 201203, China; 12023022@shutcm.edu.cn (T.G.); nieyuhan_27@163.com (Y.N.)

**Keywords:** diabetes mellitus, acupuncture therapy, traditional Chinese medicine, bibliometrics, research trends

## Abstract

**Background:** As diabetes mellitus continues to escalate into a global health crisis, particularly in China, the limitations of conventional pharmacotherapy underscore the need for complementary interventions. This study systematically reviews two decades of research progress on acupuncture for diabetes management. **Methods:** A total of 391 publications met the inclusion criteria from the Web of Science Core Collection (2004–2024) using the search terms “acupuncture” AND “diabetes”. These comprised 294 original studies and 97 reviews. CiteSpace 6.3.R1 was used to perform multidimensional analyses, including co-occurrence networks, centrality algorithms, and silhouette metrics across countries/regions, institutions, authors, journals, references, and keywords. **Results:** The analysis shows a significant increase in publications on acupuncture for diabetes management after 2013. China and the United States lead in research output, yet collaboration between the two countries remains limited. Most researchers currently work within isolated clusters, underscoring the need for greater exchanges and cooperation. Furthermore, this study identified three key research hotspots: insulin resistance, complications, and interdisciplinary research. **Conclusions:** This bibliometric analysis reveals dynamic growth patterns and paradigm shifts in acupuncture and diabetes research. The findings provide valuable implications for integrating acupuncture into diabetes treatment.

## 1. Introduction

Diabetes mellitus, as a group of metabolic diseases caused by multiple causes and characterized by chronic hyperglycemia, is based on the defects of insulin secretion and/or action [1]. This long-term hyperglycemic state not only poses a threat to an individual’s quality of life but also leads to chronic damage and dysfunction of various tissues, especially the eyes, kidneys, heart, blood vessels, and nerves [2]. According to the latest data from the International Diabetes Federation (IDF), the global diabetic population has reached 537 million, with China accounting for over 140 million cases—the highest worldwide, and this number is projected to increase by 46% by 2045 [3,4]. With changing lifestyles and accelerated population aging, the onset age of diabetes is trending younger, making it a pressing challenge in global public health. Current treatments primarily rely on hypoglycemic agents and statins, but their long-term use is associated with adverse effects such as gastrointestinal reactions, hepatic toxicity, and drug resistance, thereby increasing clinical risks [5]. Consequently, there is increasing interest in exploring complementary and potentially safer therapeutic strategies.

In Traditional Chinese Medicine (TCM), diabetes is classified under “Consumptive Thirst Disease” [6]. According to the new *International Traditional Chinese Medicine Guideline for Diagnostic and Treatment Principles of Diabetes*, diabetes is divided into two major categories: *Pi Dan* and *Xiao Dan*. Its development usually progresses through four stages: stagnation, heat, deficiency, and injury, and each stage can be further classified into different TCM syndromes [6,7]. In clinical practice, TCM diagnosis and treatment of diabetes emphasize “syndrome differentiation and treatment based on syndrome differentiation” [8]. Doctors comprehensively analyze patients’ symptoms, tongue appearance, pulse condition, and other signs to determine the specific syndrome type, and then adopt personalized treatment plans, which may include herbal medicine, acupuncture, moxibustion, massage, etc. Among these therapies, acupuncture has gained increasing attention as a complementary approach to diabetes management due to its potential efficacy and favorable safety profile [9,10]. Research suggests that acupuncture may exert its benefits by modulating glucose metabolism, hormonal balance, pancreatic function, and the central nervous system [11,12]. Furthermore, acupuncture may also alleviate complications such as vasculopathy and neuropathy [9]. However, more high-quality clinical trials are warranted to confirm these effects and elucidate the underlying mechanisms.

The purpose of this study is to systematically review the research progress on acupuncture for diabetes treatment, explore its mechanisms of action, and provide insights into future research directions. A bibliometric approach is employed for statistical and quantitative analysis. Bibliometrics is an interdisciplinary science that utilizes mathematical and statistical techniques to quantitatively analyze knowledge carriers. This approach enables the assessment of established and emerging research directions through analyses such as co-authorship, co-citation, and co-occurrence within the research field, thereby revealing research trends and knowledge structures. By focusing on the role of acupuncture and moxibustion in the treatment of diabetes, this study aims to promote the broader application and development of acupuncture in diabetes management.

## 2. Materials and Methods

### 2.1. Data Source and Search Strategy

Data for this study were sourced from the Web of Science Core Collection (WOSCC; https://webofscience.clarivate.cn/wos/alldb/basic-search; accessed on 5 December 2024) using a comprehensive search strategy designed to capture all relevant studies on acupuncture and diabetes. The search combined two conceptual categories: terms related to acupuncture interventions and terms related to diabetes. The selection of search terms was designed to maximize retrieval sensitivity through a multi-step process. First, we identified core terms from key review articles and Medical Subject Headings (MeSH) descriptors. Second, we expanded the term list by including synonyms and related phrases to cover the broad spectrum of terminology used in the literature. The search was conducted across the full text to maximize sensitivity, meaning that potentially relevant articles, particularly those in which key terms such as “acupuncture” or “diabetes” may not appear in the title or abstract but are discussed substantively in the body of the text, could still be captured. The full Boolean logic search formula, including specific keywords and syntax, is provided in Table 1.

**Figure 1:**
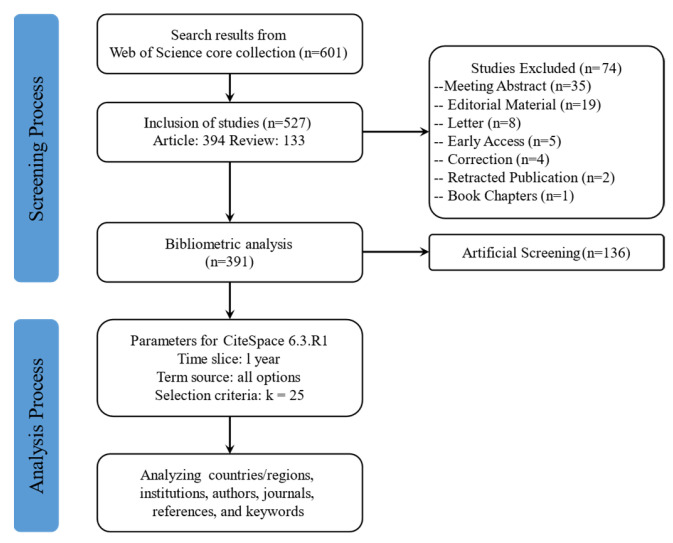
Flow chart of data processing. The initial retrieval identified 601 publications; after applying the exclusion criteria, 527 publications remained (74 excluded); following artificial screening, 391 publications were included for bibliometric analysis (136 further excluded).

Initially, 601 publications were retrieved. The inclusion criteria were as follows: the publication time frame spanned from 1 January 2004 to 5 December 2024; document types were restricted to “articles” and “reviews”; and the language was limited to “English”. After applying these criteria, 527 publications were deemed eligible. A subsequent manual screening was performed to refine the initially retrieved results. We defined a study as having “acupuncture” or “diabetes” as the core focus when at least one of the following applied: (1) the primary aim explicitly evaluated acupuncture for the prevention, treatment, mechanisms, or outcomes of diabetes or diabetic complications; (2) the principal intervention under investigation was acupuncture (or acupuncture combined with clearly described adjunct therapy) and diabetes (or diabetic outcomes) was the primary disease or endpoint; (3) the main results and conclusions centered on the effects of acupuncture in people with diabetes. We excluded studies in which “acupuncture” or “diabetes” appeared in the text but were not the core focus of the study. For example, studies in which diabetes was merely an observational parameter, while the primary focus was on evaluating the efficacy of acupuncture for stroke, were excluded [13]. Each title and abstract was independently reviewed by two researchers to assess relevance based on predefined inclusion and exclusion criteria. Discrepancies were resolved through discussion or consultation with a third reviewer. Inter-rater reliability was excellent (Cohen’s κ = 0.83; 95% CI 0.78–0.88). The data processing workflow is illustrated in Figure 1.

### 2.2. Data Collection and Analysis

Data deduplication was performed using the built-in functionality of CiteSpace 6.3.R1. CiteSpace, a Java-based application, is specifically designed to visualize and analyze evolving trends in published literature [14]. CiteSpace was employed to conduct co-occurrence and cluster analyses of the publications across various dimensions, including countries/regions, institutions, authors, journals, references, and keywords. This approach aimed to provide a comprehensive and nuanced understanding of the current landscape of the field and to identify emerging research frontiers and hotspots.

The specific parameters used in CiteSpace were as follows: the time slicing was set from 2004 to 2024, with a 1-year slice; term sources included titles, abstracts, author keywords, and keywords plus. The node types selected varied depending on the analysis purpose (e.g., “Author” for co-authorship analysis, “Keyword” for hotspot detection). The pruning method employed was “Pathfinder”, which is commonly used to simplify networks and highlight key connections.

The visualizations generated by CiteSpace consist of nodes and connecting lines represented as concentric circles. The diameter of the circles corresponds to the level of activity for a given unit, while the thickness of the lines reflects the strength of connectivity. Color variations signify temporal changes, with red nodes indicating frequent bursts of activity. Concentric circles with purple borders denote high centrality, reflecting substantial influence and robust collaboration. Nodes with a centrality value exceeding 0.1 are considered pivotal within the research domain [15].

## 3. Results

### 3.1. Analysis of Publications

**Figure 2:**
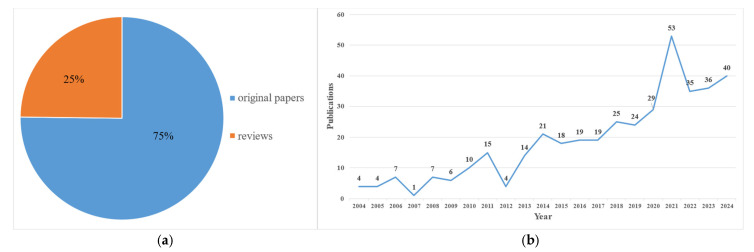
(**a**) Proportion of publications by type, indicating 294 original articles (75%) and 97 reviews (25%). (**b**) Annual publication trends in studies on acupuncture for the treatment of diabetes. The horizontal axis of the chart represents the year, and the vertical axis represents the number of publications.

A total of 391 research-related publications were ultimately included, comprising 294 original studies and 97 reviews, as shown in Figure 2a. The annual distribution of publications from 2004 to 2024 is illustrated in Figure 2b. Notably, publication output showed a slight increase from 2004 to 2006, with a total of 15 publications. The lowest publication count was recorded in 2007, with only one article. However, from 2008 onward, there was a sustained increase, reaching 15 publications in 2011, followed by a decline in 2012. After 2013, a significant upward trend emerged, culminating in 53 publications in 2021. This growth continued from 2022 to 2024, with a total of 111 publications during this three-year period.

### 3.2. Analysis of Countries/Regions

**Figure 3:**
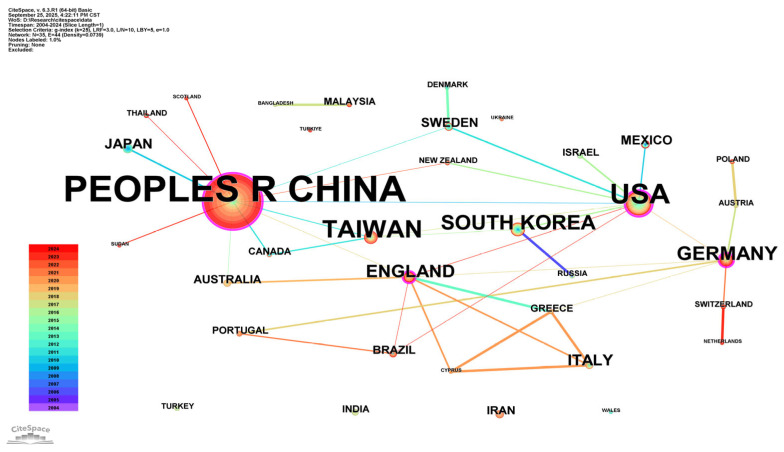
Visualization of national collaborative analysis. The nodes in the figure represent individual countries/regions, the size of the nodes corresponds to the publication output by each county/region, and the line thickness indicates the strength of cooperation between countries/regions. The purple outer rings indicate high centrality, and the node colors represent different publication years.

From 2004 to 2024, 35 countries and regions around the world contributed to research on acupuncture for the treatment of diabetes. These contributions are visually represented in Figure 3, which comprises 35 nodes and 44 connecting lines. Notably, China and the United States (USA) emerged as the most prolific contributors, reflecting their leading roles in this field. Other countries among the top 10 include South Korea, Germany, the United Kingdom, Italy, Japan, Sweden, and Mexico, none of which published more than 20 publications. In the network visualization, the United States exhibited the highest centrality value (0.52), followed by China (0.45) and Germany (0.38).

The size of the nodes corresponds to the number of publications from the respective country, and the thickness of the lines indicates the strength of collaboration between countries. Notably, China demonstrated stronger collaborative ties with the United States, Taiwan (China), and Germany. However, as shown in the figure, although cooperation exists between China and the United States, it remains limited in scope, with most collaborative activities occurring in earlier years. Both China and the United States appear to prioritize cooperation with their respective neighboring countries.

### 3.3. Analysis of Major Institutions

**Figure 4:**
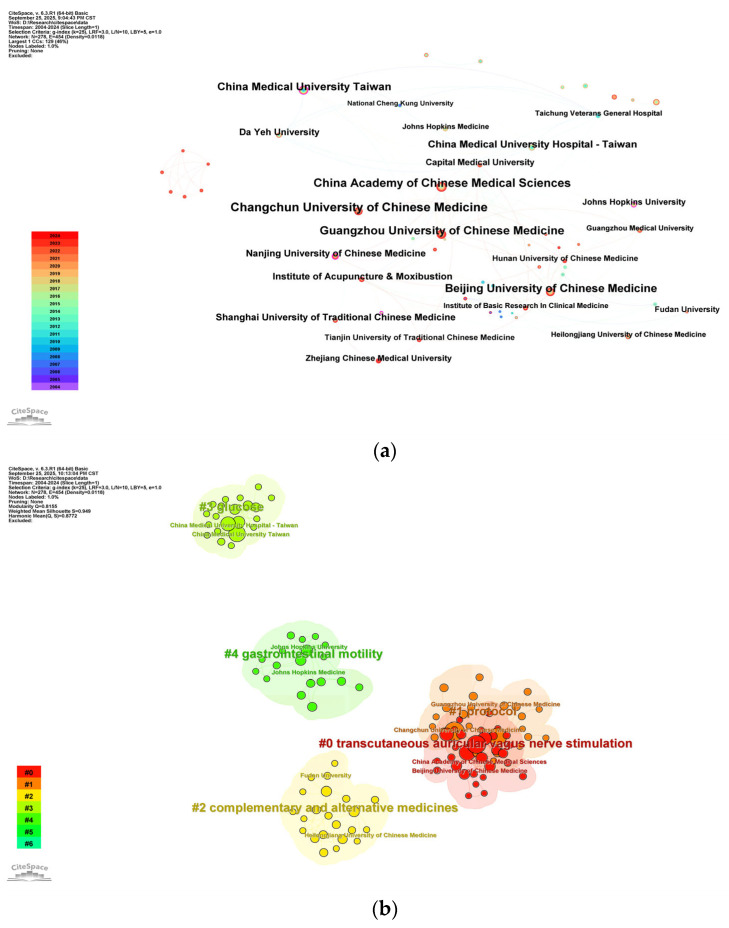
(**a**) Visualization of collaborative analyses of institutions. All institutions shown have published at least 5 publications. The nodes in the figure represent a single institution, the size of the nodes corresponds to the publication output by each institution, and the line thickness indicates the strength of the cooperation between institutions. The purple outer rings indicate high centrality, and the node colors represent different publication years. (**b**) Keyword cluster analysis of institutional research topics. Only the first 5 clusters are shown, with each cluster representing institutions that have published at least 5 publications, each node representing one institution, different color groups representing different clusters, and text with # representing keyword clusters for these institutions, #2 “Complementary and alternative medicines”.

From 2004 to 2024, a total of 278 institutions actively contributed to research on acupuncture for diabetes treatment. A detailed analysis of these institutions was conducted using visual mapping, as shown in Figure 4a, which revealed 278 nodes and 454 connecting lines. Table 2 lists the top 10 institutions by publication output, with Changchun University of Chinese Medicine ranking first (28 publications), followed by the China Academy of Chinese Medical Sciences (25 publications) and Guangzhou University of Chinese Medicine (23 publications). Regarding institutional centrality, Nanjing University of Chinese Medicine ranked highest with a centrality score of 0.16, followed by China Medical University in Taiwan (0.12), and both the China Academy of Chinese Medical Sciences and Beijing University of Chinese Medicine (each with 0.08). The institutional co-occurrence analysis revealed that the node sizes in the graph correspond to the number of original publications from each institution, while the line thickness represents the strength of collaboration between institutions. The findings indicate that several institutions, including the Chinese Academy of Medical Sciences, Changchun University of Chinese Medicine, Guangzhou University of Chinese Medicine, Beijing University of Chinese Medicine, China Medical University in Taiwan, the China Institute of Acupuncture and Moxibustion, Shanghai University of Chinese Medicine, and Nanjing University of Chinese Medicine, have established extensive collaborative networks.

To further explore cross-institutional research themes, a cluster analysis of keywords from institutional publications was performed. The analysis identified six primary clusters, represented by distinct color regions in Figure 4b, with high confidence values (Q = 0.8155, S = 0.949). The largest cluster, #0, was labeled “transcutaneous vagal nerve stimulation,” followed by #1 “medical protocols” and #2 “complementary and alternative medicines.” Notably, the top three institutions demonstrated a focus on specific thematic areas: #0 “transcutaneous vagal nerve stimulation,” #1 “medical protocols,” and #3 “glucose,” highlighting their unique contributions to these research domains.

### 3.4. Analysis of Authors and Co-Cited Authors Analysis

**Figure 5:**
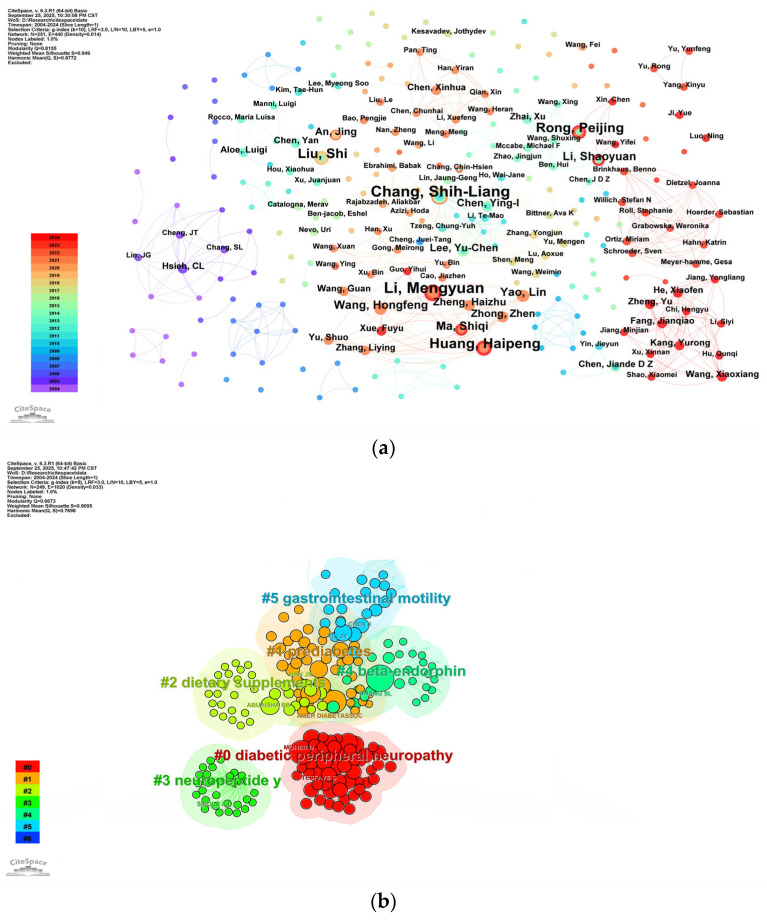
(**a**) Visualization of collaborative analysis of authors. Each node represents an author, the size of the nodes corresponds to the publication output by each author, and the line thickness indicates the strength of cooperation between authors. The purple outer rings indicate high centrality, and the node colors represent different publication years. (**b**) Keyword cluster analysis of co-cited authors’ research topics. Only the top 6 clusters are shown. Each node represents an author, different color groups represent different clusters, and text with # indicates keyword clusters of these co-cited authors, #2 = dietary supplements.

Between 2004 and 2024, a total of 251 researchers actively contributed to research on acupuncture for diabetes. A co-occurrence analysis, presented in Figure 5a, mapped the publication output and collaboration patterns among these researchers, revealing 251 nodes and 440 connecting links. Table 3 presents the top 10 authors ranked by publication volume. Notably, the productivity was relatively low (the most prolific author published only 10 publications), and the top contributors (such as Chang Shih-Liang and Li Meng-Yuan) exhibited low centrality, suggesting a lack of strong collaborative partnerships among leading research groups.

Co-citation analysis identified the foundational literature and key researchers shaping the field’s intellectual foundation (Table 3). Chang S.L. was both the most cited (43 citations) and one of the most central authors, alongside Abuaisha B.B. and the American Diabetes Association. The robust cluster analysis (Q = 0.6673, S = 0.9095) of these co-cited authors’ keywords, as visualized in Figure 5b, further confirms that research focuses on topics such as diabetic peripheral neuropathy and neuropeptide mechanisms. The cluster analysis revealed that research themes primarily encompass diabetes-related areas, including prediabetes and diabetic peripheral neuropathy (DPN), as well as other domains such as neuropeptide Y, beta-endorphin, and gastrointestinal motility. This analysis provides valuable insights into current research hotspots and offers guidance for future researchers to pursue targeted studies within their respective domains.

### 3.5. Analysis of Journals and Co-Cited Journals Analysis

Among publications examining acupuncture for diabetes, the most frequently cited articles were published in prestigious journals such as *The Lancet* and *The New England Journal of Medicine*. Additionally, journals including *Diabetes* and *Diabetes Research and Clinical Practice* have played pivotal roles in elucidating the mechanisms of acupuncture in diabetes treatment. A co-citation analysis of these journals underscores their authority and influence in the field. Notably, seven journals each garnered over 100 citations. Table 4 lists the top 10 most frequently cited journals, with *Diabetes Care* ranking first, followed by *Evidence-Based Complementary and Alternative Medicine* and *Acupuncture in Medicine*. Regarding centrality among co-cited journals, *Diabetes Care*, *Evidence-Based Complementary and Alternative Medicine*, and *Neuroscience Letters* shared the top position, each with a centrality score of 0.15.

### 3.6. Analysis of Co-Cited References and Reference Burst

**Figure 6:**
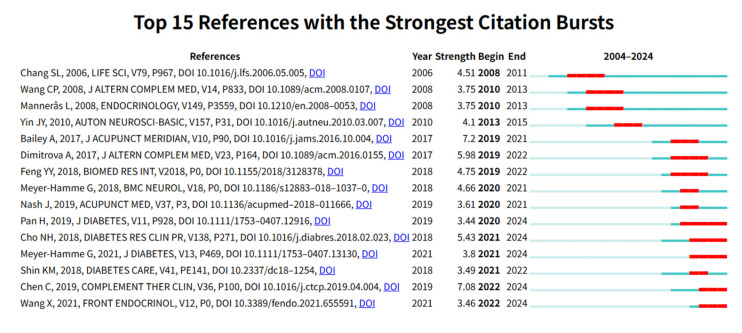
The top 15 most-cited publications [9,16,17,18,19,20,21,22,23,24,25,26,27,28,29].

A detailed analysis of the co-cited references and citation bursts was conducted, as summarized in Table 5. Notably, the study by Chen, C. et al. received the highest number of citations (*n* = 19). The top 10 cited publications primarily focused on Type 2 diabetes mellitus (T2DM) and its complications, such as DPN and polycystic ovary syndrome. The main interventions discussed included acupuncture, electroacupuncture, and TCM techniques such as acupoint application. Figure 6 presents the results of reference burst analysis. Higher burst strength values indicate a sharp increase in citations for specific publications within a given period. Among these, the study by Pan, H. et al. exhibited the highest burst strength. This research focused on evaluating the preventive effects of electroacupuncture therapy, specifically the “adjusting internal organs and dredging channel” approach, on the progression of DPN in rats. Additionally, studies by Pan, H. et al., Cho, N.H. et al., and Meyer-Hamme, G. et al. demonstrated citation bursts of long duration, indicating sustained academic influence within the research community. Notably, Meyer-Hamme, G. et al., and Wang, X. et al. represent some of the most recent studies within the past five years. These studies provide a robust scientific foundation for applying acupuncture in the treatment of DPN and highlight the potential of acupuncture in improving nerve function and enhancing the quality of life for patients, representing significant advances in this field of research.

### 3.7. Analysis of Frontiers and Hotspots

**Figure 7:**
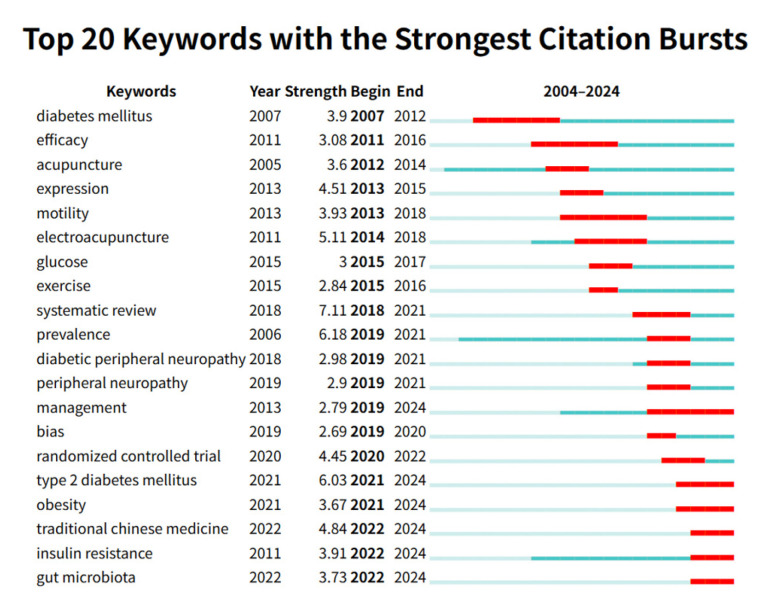
The top 20 keywords with the strongest citation bursts.

Keywords serve as key indicators of research focus within a field. Figure 7 presents a burst analysis of the top 20 keywords in acupuncture for diabetes research. Burst strength is calculated based on both keyword frequency and temporal patterns, with higher values indicating a significant increase in the prevalence of a keyword during a specific period. Notably, the keywords with the longest burst durations were “diabetes mellitus,” “efficacy,” and “management,” suggesting that diabetes management and treatment efficacy remain areas of sustained research interest. Keywords that have emerged prominently in the last five years include “randomized controlled trial,” “type 2 diabetes mellitus,” “obesity,” “traditional Chinese medicine,” “insulin resistance,” and “gut microbiota,” indicating that these topics currently represent cutting-edge research directions in this field.

**Figure 8:**
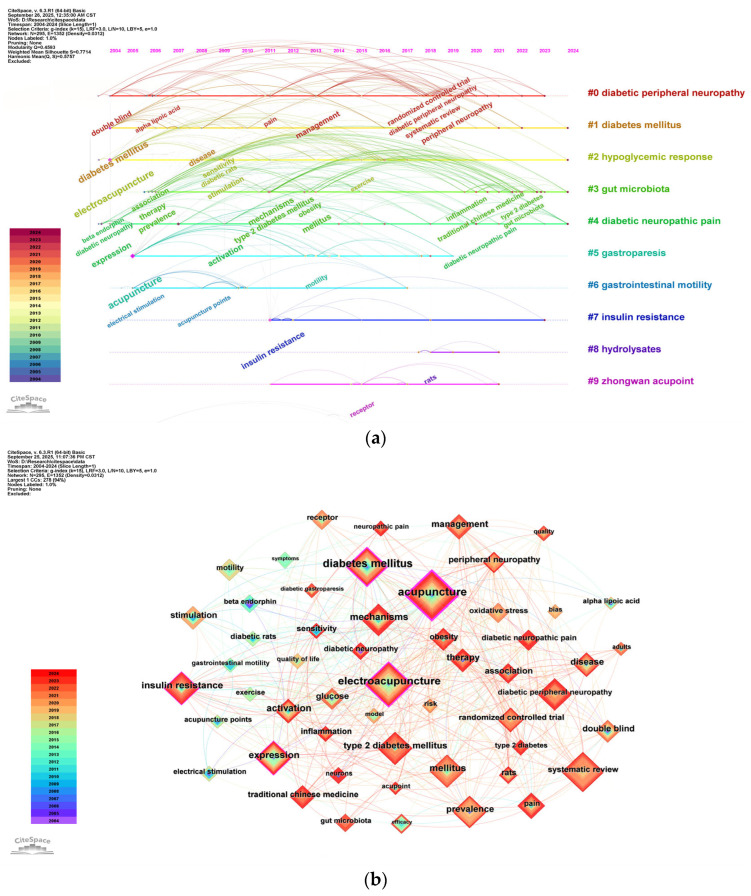
(**a**) Timeline visualization of keyword cluster analysis. The top 10 clusters are shown, with the cluster labels arranged sequentially on the right. Keywords on the same horizontal timeline belong to the corresponding cluster. Keywords are represented by nodes along the timeline; the size of the nodes corresponds to the frequency of keyword occurrence, and the node position along the horizontal axis indicates the year of the keyword’s first appearance. (**b**) Co-occurrence network of keywords. Keywords shown appear more than 10 times. Each node represents a keyword. The size of the nodes is proportional to occurrence frequency, and the line thickness indicates the strength of co-occurrence between keyword pairs. The node colors represent different years of the keyword occurrence.

Following this, a cluster analysis was subsequently performed on all keywords to track the evolution of research topics in acupuncture treatment for diabetes over time. After conducting a timeline analysis of keywords using CiteSpace, Figure 8a presents the top 10 clusters. This analysis involved 295 nodes and 1352 links, with Q = 0.4593 and S = 0.7714, indicating a significant cluster structure and credible results. The names of the clusters are displayed in order on the right, with keywords on the same horizontal line belonging to the same cluster. Each keyword is represented by a node on the horizontal line, and the size of the node corresponds to the frequency of keyword occurrence. The thickness of the connecting lines between nodes reflects the strength of keywords co-occurring. The timeline analysis reveals that research has predominantly focused on diabetes treatment, its complications, therapeutic mechanisms, and acupuncture’s role in managing these complications, which have attracted growing scholarly attention. Figure 8b illustrates the co-occurrence of keywords. The keyword “acupuncture” appears most frequently, with 71 occurrences, followed by “electroacupuncture,” “diabetes mellitus,” and “systematic review,” all appearing more than 30 times. Table 6 lists the top 20 keywords by frequency, representing the central themes in acupuncture research for diabetes treatment.

**Figure 9:**
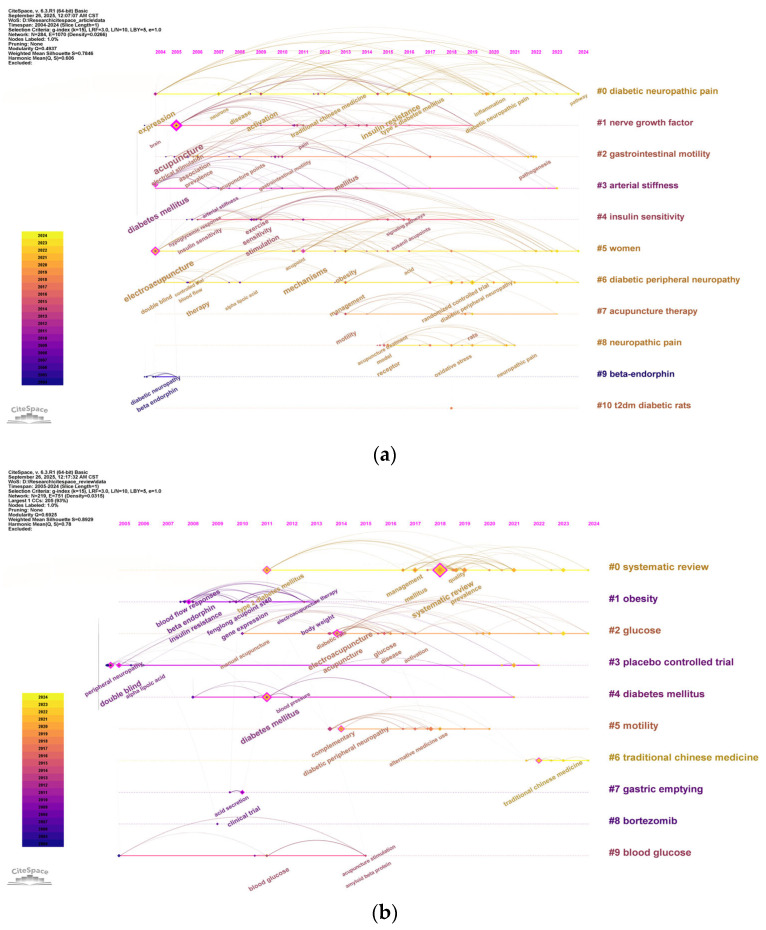
(**a**) Timeline visualization of keyword cluster analysis for original research articles. The top 11 clusters are shown. (**b**) Timeline visualization of keyword cluster analysis for review articles. The top 10 clusters are shown. The cluster labels are displayed sequentially on the right, with keywords on the same horizontal timeline belonging to the respective clusters. Keywords are represented by nodes along the timeline; the size of the nodes corresponds to the frequency of keyword occurrence, and the node position along the horizontal axis indicates the year of the keyword’s first appearance.

A hierarchical analysis was performed by combining original studies and reviews, which revealed distinct keyword clusters. For original research articles, the keyword timeline analysis, shown in Figure 9a, identified the top 11 clusters. The analysis showed that original research has primarily focused on neuropathic pain, nerve growth factors, gastrointestinal motility, arteriosclerosis, insulin sensitivity, DPN, acupuncture therapy, and related topics. Additionally, studies have investigated how acupuncture treatment can improve insulin sensitivity and exert anti-inflammatory effects in T2DM, providing mechanistic insights into these processes. The impact of acupuncture on diabetes through gut microbiota regulation has also been explored, laying the groundwork for integrating TCM with modern diabetes management strategies. The keywords timeline analysis for reviews, shown in Figure 9b, identified the top 10 clusters. These reviews mainly focus on diabetes management, obesity, gastric motility, glucose control, placebo-controlled experiments, Chinese medicine, gastric emptying, and the use of bortezomib and other drugs. These topics encompass various aspects of diabetes treatment, including medication, lifestyle management, and clinical trial design.

## 4. Discussion

### 4.1. General Information

The analysis of basic data reveals a consistent upward trend in research on acupuncture for diabetes, particularly after 2013. By 2021, the annual publication count reached 53, reflecting growing academic interest and an expanding research base.

The dataset comprises 294 original studies and 97 reviews. China is the largest contributor (240 publications), followed by the United States (59). The collaboration network shows structural contradictions: China’s partnerships are geographically dispersed rather than strategically deep, with limited collaborative output with the United States, though stronger links exist with Scotland, Thailand, Sudan, Switzerland, and the Netherlands. Given the varying strengths and advantages of different nations, there is a pressing need to expand the scope and depth of international collaboration in this increasingly globalized field. Such efforts could create a conducive environment for achieving more impactful research outcomes and increasing publication output.

In the analysis of major institutions, Changchun University of Chinese Medicine has emerged as a prominent institution in this field, with a robust record of publications focusing primarily on the potential of acupuncture to alleviate diabetes-related complications [19,30,31,32,33,34,35,36]. Among its contributions, the most-cited study investigated the mechanisms of acupuncture in treating DPN. This study demonstrated that electroacupuncture effectively improved peripheral neuropathy in streptozotocin-induced diabetic rats by reducing the expression of glucose-regulated protein 78 (GRP78) and caspase-12, thereby mitigating sciatic nerve damage [19].

Co-citation analysis identifies ABUAISHA BB (0.33) as the author with the highest centrality, while the top 10 productive authors show limited network centrality, with publication counts clustered between 5 and 8. This “high output, low connectivity” paradox suggests isolated research clusters rather than true collaboration. To address this, authors are encouraged to broaden their collaborative networks, foster knowledge exchange, and enhance academic output.

The journals with the highest publication volumes include *Diabetes* and *Diabetes Research and Clinical Practice*, both of which have been instrumental in elucidating treatment strategies and mechanisms of acupuncture for diabetes. Among co-cited journals, *Diabetes Care* is the most frequently cited. The most-cited study from these journals is a systematic review and meta-analysis of randomized controlled trials (RCTs) on acupuncture for T2DM [9]. This study concluded that acupuncture can be recommended as an adjunctive treatment for T2DM, particularly for obese or metabolically challenged patients. Citation and keyword analysis further reveal the interdisciplinary nature of this field, incorporating TCM, modern medicine, anatomy, biostatistics, imaging, neurology, and microbiology. This multifaceted approach facilitates a comprehensive understanding of the mechanisms underlying acupuncture’s effects on diabetes, thereby paving the way for more effective treatment strategies to benefit patients.

### 4.2. Hot Topics and Frontiers

We employed CiteSpace software to perform keyword clustering and outbreak analysis, thereby uncovering the research hotspots and frontier trends in the field of acupuncture treatment for diabetes. The cluster and burst analyses of keywords collectively illustrate the dynamic research landscape in this domain. Cluster analysis offers a macroscopic overview of the research topics, while keyword burst analysis accentuates research hotspots within specific timeframes. The integration of both methods provides a comprehensive perspective for identifying and forecasting future research trends.

Clusters #1 diabetes mellitus and #7 insulin resistance are directly pertinent to the mechanisms by which acupuncture influences diabetes and its insulin resistance. Clusters #0 diabetic peripheral neuropathy, #4 diabetic neuropathic pain, and #5 gastroparesis highlight the application of acupuncture in managing complications such as diabetic neuropathy, neuralgia, and gastroparesis. Clusters #3 gut microbiota and #9 zhongwan acupoint offer insights into how acupuncture affects diabetes through modulation of gut microbiota and the action of specific acupoints. The keyword burst analysis further corroborates these findings.

The outbreak analysis revealed that “systematic review” exhibited the highest outbreak intensity between 2018 and 2021, underscoring the significance of conducting comprehensive reviews of existing research on acupuncture for diabetes. Most systematic reviews in this field include RCTs, where the intervention group receives standardized acupuncture (e.g., ST36, SP6, CV12), and the control group receives sham acupuncture, placebo, or conventional treatment. Primary endpoints include fasting blood glucose (FBG), HbA1c, Homeostasis Model Assessment of Insulin Resistance (HOMA-IR), and symptom scores.

Additionally, the prolonged outbreak duration of “management” indicates sustained attention to diabetes management. Meanwhile, “type 2 diabetes mellitus,” “obesity,” “traditional Chinese medicine,” “insulin resistance,” and “gut microbiota” are ongoing concerns. The outbreak of these keywords highlights the necessity for in-depth research on acupuncture’s role in treating T2DM and its underlying mechanisms, particularly in enhancing insulin sensitivity and anti-inflammatory effects. The long-term outbreak duration of the keyword “management” and concerns regarding “type 2 diabetes mellitus” and “obesity” suggest the potential role of acupuncture in managing diabetes and associated complications. The ongoing focus on “gut microbiota” and “traditional Chinese medicine” provides a basis for exploring how acupuncture influences diabetes through the regulation of gut microbiota and how TCM can be integrated with modern diabetes management strategies. Many of these studies are prospective clinical trials or cohort studies enrolling T2DM patients, with intervention durations ranging from 4 to 12 weeks. Outcomes typically include metabolic parameters, inflammatory biomarkers, and gut microbiota composition.

Acupuncture has been incorporated into the Chinese Guidelines for the Prevention and Treatment of T2DM (2020 edition) [37]. Existing research has demonstrated that acupuncture and moxibustion exert their therapeutic effects on diabetes through multiple mechanisms, including the regulation of blood glucose and hormone levels [38,39,40], enhancement of insulin sensitivity [24,41], modulation of the central nervous system [42], and improvement of lipid metabolism [20,43]. These mechanisms collectively contribute to improving the metabolic status of diabetic patients and reducing the risk of complications. Furthermore, acupuncture has shown efficacy in managing diabetes-related complications such as neuropathy [44], nephropathy [33], and cardiovascular disease [45]. This part of human clinical research usually adopts pre- and post-treatment or randomized controlled designs, and the treatment duration is 6 to 12 weeks. Endpoints include serum biomarkers, quality of life assessments, and nerve conduction velocities. Additionally, interdisciplinary research is crucial for advancing the field of acupuncture treatment for diabetes. This includes investigating the impact of different acupoint combinations on therapeutic outcomes 17, utilizing advanced imaging technologies [46], and exploring the neurobiological and microbiological mechanisms underlying acupuncture’s effects [47,48]. Such research may provide novel perspectives and approaches for diabetes management.

## 5. Strengths and Limitations

This study provides seminal contributions to the bibliometric analysis of acupuncture for diabetes. By comprehensively examining literature spanning 2004 to 2024, we identified key research trends and hotspots, offering actionable insights to inform future investigations. A primary strength lies in the extensive 20-year scope, which establishes a solid data foundation. CiteSpace-aided visualization further elucidated the field’s developmental trajectory, structural relationships, and emerging trends.

However, several limitations must be acknowledged. First, the limited number and relatively low methodological rigor of existing studies on acupuncture for diabetes may have constrained analytical depth and the robustness of bibliometric inferences. Second, exclusive reliance on WOSCC data potentially restricted publication coverage, affecting conclusion robustness. Search term selection may have introduced subjectivity. Although CiteSpace enhanced analytical efficiency, its inherent limitations (e.g., algorithm biases) may have compromised result accuracy. Additionally, the absence of rigorous quality assessment for included literature risks outcome bias. Time lags in publishing highly cited works may also have skewed citation rate evaluations. Lastly, the 2004–2024 timeframe limits the assessment of longer-term trends.

To address these limitations, future bibliometric studies should aim to broaden database coverage by incorporating more comprehensive international and regional sources. Furthermore, employing multiple bibliometric tools and conducting cross-platform comparisons could help reduce tool-specific biases and strengthen the robustness of the findings. It is also important to note that bibliometric indicators, such as citation counts and keyword frequencies, reflect patterns of academic attention and discourse rather than direct measures of clinical effectiveness or practical impact.

## 6. Conclusions

This bibliometric analysis of acupuncture applications in diabetes management (2004–2024) revealed a significant increase in publication output, with China and the United States leading research productivity despite limited international collaboration. Most experts in the field conduct independent research, indicating a need for enhanced academic exchanges. The study identified key research hotspots, including insulin modulation mechanisms, complication-specific pathways, and cross-domain synergies. To further advance the field, future research should broaden the scope of databases and employ a diverse array of bibliometric tools to enable comprehensive analyses based on an increasing number of studies. While this study illustrates important trends in acupuncture research related to diabetes management, it is crucial to emphasize that these findings reflect research trends rather than definitive claims of clinical efficacy. Continued exploration is warranted to fully understand acupuncture’s potential contributions to global efforts in diabetes management.

## Figures and Tables

**Table 1:** The search formula.

Number	Contents
#1	TS = (“Acupuncture” OR “Pharmaco acupuncture Treatment” OR “Electroacupuncture” OR “Electro-acupuncture” OR “Body Acupuncture” OR “Manual Acupuncture” OR “Auricular” OR “Auricular Acupuncture” OR “Auricular Needle” OR “Acupuncture Point” OR “Ear Acupuncture” OR “Warm Acupuncture” OR “needle warming moxibustion” OR “Moxibustion” OR “moxibustion” OR “Thermal moxibustion” OR “Acupoint Injection” OR “Catgut Embedding” OR “Catgut Implantation at Acupoint” OR “Embedding Thread” OR “Dry needle” OR “transcutaneous electrical acupoint stimulation” OR “Scalp acupuncture” OR “fire needle”)
#2	TS = (“Diabetes mellitus” or “Diabetes *” or “Diabetic *” or “Diabetic mellitus”)
#3	#1 AND #2

* Note: “*” is the truncation symbol; it expands a word stem to any ending.

**Table 2:** Top 10 countries/regions and institutions by publication count.

Items	Rank	Name	Centrality	Year *	Publications
Country/region	1	People’s Republic of China	0.45	2008	240
	2	United States of America	0.52	2004	59
	3	Taiwan (China)	0.04	2004	34
	4	South Korea	0.09	2004	17
	5	Germany	0.38	2006	14
	6	England	0.3	2011	11
	7	Italy	0	2010	8
	8	Japan	0	2009	6
	9	Sweden	0.09	2008	5
	10	Mexico	0	2007	5
Institution	1	Changchun University of Chinese Medicine	0.02	2009	28
	2	China Academy of Chinese Medical Sciences	0.08	2008	25
	3	Guangzhou University of Chinese Medicine	0.1	2014	23
	4	Beijing University of Chinese Medicine	0.08	2013	21
	5	China Medical University Taiwan	0.12	2005	20
	6	China Medical University Hospital—Taiwan	0	2008	14
	7	Huazhong University of Science and Technology	0	2011	13
	8	Institute of Acupuncture and Moxibustion	0.01	2008	12
	9	Shanghai University of Traditional Chinese Medicine	0.03	2011	12
	10	Nanjing University of Chinese Medicine	0.16	2013	12

* Note: Year indicates the earliest year when the country/region or institution appeared in the dataset.

**Table 3:** Top 10 authors and co-authors by publication count.

Items	Rank	Name	Centrality	Year *	Publications/Count
Author	1	Chang, Shih-Liang	0	2006	10
	2	Li, Meng-Yuan	0	2021	10
	3	Huang, Hai-Peng	0	2021	8
	4	Rong, Pei-Jing	0	2014	7
	5	Liu, Shi	0	2013	7
	6	Li, Shao-Yuan	0	2014	5
	7	Wang, Hong-Feng	0	2021	5
	8	Yao, Lin	0	2021	5
	9	Ma, Shi-Qi	0	2021	5
	10	Lee, Yu-Chen	0	2011	4
Co-author	1	Chang, S.L.	0.31	2004	43
	2	Moher, D.	0.01	2019	32
	3	Amer Diabet Assoc	0.12	2013	31
	4	Liang, F.	0.04	2014	21
	5	Tesfaye, S.	0.04	2020	18
	6	Garrow, A.P.	0.04	2019	17
	7	Chen, W.	0.03	2014	16
	8	Boultion, A.J.M.	0.05	2005	16
	9	Abuaisha, B.B.	0.33	2004	16
	10	Li, J.	0.01	2021	15

* Note: Year indicates the earliest year when the authors and co-authors published an article in the dataset.

**Table 4:** Top 10 most co-cited journals.

Rank	Co-Cited Journal	Year *	Count	Centrality	IF (2024)	JCR
1	Diabetes Care	2006	181	0.15	14.8	Q1
2	Evidence-Based Complementary and Alternative Medicine	2011	156	0.15	2.65	Q3
3	Acupuncture in Medicine	2006	142	0.11	2.4	Q2
4	PLoS One	2013	139	0.12	2.9	Q1
5	Diabetologia	2004	123	0.13	8.4	Q1
6	Diabetes Research and Clinical Practice	2006	106	0.05	6.1	Q1
7	Diabetes	2009	101	0.13	6.2	Q1
8	Journal of Alternative and Complementary Medicine	2010	86	0.06	2.3	Q2
9	American Journal of Chinese Medicine	2008	83	0.08	4.8	Q1
10	Neuroscience Letters	2004	79	0.15	2.5	Q3

* Note: Year indicates the earliest year when the journal began to be jointly cited in the dataset. Abbreviations: IF, Impact Factor; JCR, Journal Citation Reports.

**Table 5:** Top 10 most co-cited references.

Rank	Author	References	Citations	Centrality
1	Chen, C.	Acupuncture for type 2 diabetes mellitus: A systematic review and meta-analysis of randomized controlled trials [9].	19	0.01
2	Cho, N.H.	IDF Diabetes Atlas: Global estimates of diabetes prevalence for 2017 and projections for 2045 [16].	16	0.02
3	Dimitrova, A.	Acupuncture for the Treatment of Peripheral Neuropathy: A Systematic Review and Meta-Analysis [17].	15	0.01
4	Bailey, A.	Acupuncture Treatment of Diabetic Peripheral Neuropathy in an American Indian Community [18].	15	0.01
5	Pan, H.	“Adjusting internal organs and dredging channel” electroacupuncture treatment prevents the development of diabetic peripheral neuropathy by downregulating glucose-related protein 78 (GRP78) and caspase-12 in streptozotocin-diabetic rats [19].	14	0.03
6	Feng, Y.Y.	Acupoint Therapy on Diabetes Mellitus and Its Common Chronic Complications: A Review of Its Mechanisms [20].	12	0.03
7	Meyer-Hamme, G.	ACUDIN—ACUpuncture and laser acupuncture for treatment of DIabetic peripheral Neuropathy: A randomized, placebo-controlled, partially double-blinded trial [21].	9	0
8	Meyer-Hamme, G.	Electrophysiologically verified effects of acupuncture on diabetic peripheral neuropathy in type 2 diabetes: The randomized, partially double-blinded, controlled ACUDIN trial [22].	9	0.02
9	Wang, X.	Electroacupuncture Alleviates Diabetic Peripheral Neuropathy by Regulating Glycolipid-Related GLO/AGEs/RAGE Axis [23].	8	0.01
10	Chang, S.L.	Enhanced insulin sensitivity using electroacupuncture on bilateral Zusanli acupoints (ST 36) in rats [24].	8	0.02

**Table 6:** The 20 keywords with the highest frequency.

Rank	Keyword	Centrality	Year *	Count
1	acupuncture	0.73	2005	71
2	electroacupuncture	0.25	2011	45
3	diabetes mellitus	0.33	2007	36
4	systematic review	0.1	2018	34
5	diabetic peripheral neuropathy	0.04	2018	29
6	mellitus	0.13	2013	26
7	expression	0.03	2013	21
8	insulin resistance	0.06	2011	20
9	type 2 diabetes mellitus	0.03	2021	19
10	prevalence	0.02	2006	18
11	mechanisms	0.08	2011	17
12	management	0.05	2013	17
13	activation	0.05	2009	16
14	stimulation	0.16	2009	13
15	traditional Chinese medicine	0.01	2022	11
16	randomized controlled trial	0.02	2020	11
17	glucose	0.02	2015	11
18	pain	0.03	2011	11
19	motility	0.02	2013	10
20	diabetic neuropathic pain	0.01	2020	10

* Note: Year indicates the earliest year when the keyword is referenced in the dataset.

## Data Availability

The original data presented in the study were retrieved from the WOSCC and are openly available through the search strategy detailed in the manuscript.

## Abbreviations

The following abbreviations are used in this manuscript:

DPN	Diabetic peripheral neuropathy
FBG	Fasting blood glucose
GRP78	Glucose-regulated protein 78
HbA1c	Glycated hemoglobin
HOMA-IR	Homeostasis model assessment of insulin resistance
IDF	International Diabetes Federation
MeSH	Medical Subject Headings
RCTs	Randomized controlled trials
T2DM	Type 2 diabetes mellitus
TCM	Traditional Chinese Medicine
USA	the United States
WOSCC	Web of Science Core Collection
